# Cytotoxic Drugs Activate KSHV Lytic Cycle in Latently Infected PEL Cells by Inducing a Moderate ROS Increase Controlled by HSF1, NRF2 and p62/SQSTM1

**DOI:** 10.3390/v11010008

**Published:** 2018-12-24

**Authors:** Marisa Granato, Maria Saveria Gilardini Montani, Camilla Angiolillo, Gabriella D’Orazi, Alberto Faggioni, Mara Cirone

**Affiliations:** 1 Department of Experimental Medicine, “Sapienza” University of Rome, Italy, Laboratory affiliated to Istituto Pasteur Italia-Fondazione Cenci Bolognetti, Viale Regina Elena 324, 00161 Rome, Italy; marisa.granato@uniroma1.it (M.G.); mariasaveria.gilardinimontani@uniroma1.it (M.S.G.M.); camilla_angiolillo@yahoo.it (C.A.); 2 Department of Research, Advanced Diagnostics, and Technological Innovation, Regina Elena National Cancer Institute, 00144 Rome, Italy; gdorazi@unich.it; 3 Department of Medical, Oral and Biotechnological Sciences, University “G. d’Annunzio”, 66013 Chieti, Italy

**Keywords:** KSHV lytic cycle, ROS, HSF1, NRF2, p62/SQSTM1, Bortezomib, TPA, butyrate

## Abstract

Previous studies have indicated that cytotoxic treatments may induce or not activate viral lytic cycle activation in cancer cells latently infected by Kaposi’s sarcoma-associated herpesvirus (KSHV). To investigate the molecular mechanisms responsible for such an effect, we compared two cytotoxic treatments able to induce the viral lytic cycle, named 12-O-tetradecanoylphorbol 13-acetate (TPA) (T) in combination with sodium butyrate (B) and bortezomib (BZ), with two cytotoxic treatments that did not activate this process, named metformin (MET) and quercetin (Q). Our results indicated that TB and bortezomib increased levels of oxygen reactive species (ROS) while metformin and quercetin reduced them. The finding that N-acetylcysteine (NAC), a reactive oxigen species (ROS) scavenger, counteracted K-bZIP expression induced by TB or bortezomib, confirmed that an ROS increase played a role in KSHV lytic cycle activation. Moreover, we found that TB and bortezomib up-regulated p62/Sequestosome1(p62/SQSTM1) protein, while metformin and quercetin down-regulated it. p62/SQSTM1 silencing or the inhibition of NF-E2-related factor 2 (NRF2) or Heat Shock Factor 1 (HSF1), that mediate p62/SQSTM1 transcription, also reduced KSHV lytic antigen expression induced by TB or bortezomib. Interestingly, such combination treatments further increased intracellular ROS and cytotoxicity induced by the single TB or bortezomib treatment, suggesting that NRF2, HSF1 and p62/SQSTM1 keep the ROS level under control, allowing primary effusion lymphoma (PEL) cells to continue to survive and KSHV to replicate.

## 1. Introduction

It has not been clarified why some cytotoxic treatments induce gammaherpesvirus replication in lymphoma cells harboring latent Epstein–Barr virus (EBV) or Kaposi sarcoma-associated herpesvirus (KSHV) infection. Lytic cycle activation may be considered a side effect of chemotherapies, since it leads to the expression of viral lytic antigens that may promote tumorigenesis. Particularly in the case of KSHV, viral replication and viral spread contribute to the maintenance of viral-associated malignancies [1]. Therefore, it is important to understand, at the molecular level, which pathways are activated by cytotoxic drugs that induce the viral lytic cycle in order to target them to restrain KSHV replication. In this study, we compared the effects induced by cytotoxic treatments that activate KSHV lytic cycle in primary effusion lymphoma (PEL) cells, namely 12-O-tetradecanoylphorbol 13-acetaten (TPA) in combination with sodium butyrate (TB) and bortezomib (BZ), with those mediated by metformin (MET) and quercetin (Q) that, although cytotoxic for PEL cells [2,3,4,5], did not affect viral replication. We investigated whether these drugs could influence the levels of intracellular oxygen reactive species (ROS), since these molecules have been reported to promote the KSHV lytic cycle [6]. Moreover, as we have recently shown, it is known that ROS may activate the extracellular signal-regulated kinase 1/2 (ERK1/2), which in turn phosphorylates serine 15 p53, promoting the transcription of p21 and activating KSHV replication [3]. Accordingly, previous separate findings have indicated that the activation of ERK1/2 [7] and p53-p21 axis were essential for the induction of KSHV replication in PEL cells [8]. In response to the intracellular ROS increase, transcription factors such as NF-E2-related factor 2 (NRF2) and Heat Shock Factor 1 (HSF1), the main mediators of the anti-oxidant and heat shock response, may be activated to protect cells from stress [9]. Among other molecules, these transcription factors promote the transcription of p62/Sequestosome1 (p62/SQSTM1) [10]. Notably, p62/SQSTM1 accumulation may also be the consequence of autophagy inhibition, not only because the reduction of autophagy causes cellular stress, but because autophagy represents the main route for p62/SQSTM1 degradation [11]. Interestingly, p62/SQSTM1 activates the p62/SQSTM1-KEAP1-NRF2 axis and increases NRF2 stabilization through a positive feedback loop [10]. In this study, we investigated whether the level of intracellular ROS could influence the expression of p62/SQSTM1 in TB-, BZ-, MET-, and Q-treated PEL cells, and evaluated its role in KSHV lytic cycle activation. It has been shown that p62/SQSTM1 negatively affects viral replication in the case of dengue virus [12], while its role in KSHV lytic cycle has not yet been investigated. We then explored whether HSF1 and NRF2, the main transcription factors promoting p62/SQSTM1 transcription, could also play a role in KSHV lytic cycle induced by TB or BZ. It has been reported that NRF2 inhibition may affect KSHV lytic reactivation [13] and, interestingly, HSP70, another common target of HSF1 and NRF2, seems to be essential for KSHV replication, being recruited at the replication compartments (RTCs) [14]. Since several molecular pathways, activated in response to stress to help cells to cope with it, concomitantly promote viral replication, it could be that their inhibition allows us to deal with two birds with one stone. Indeed, survival and progression of KSHV viral-associated malignancies are also dependent on continuous viral release and renewal of viral infection [15]. Based on these considerations, the aim of this study was to investigate which molecules, and/or molecular pathways, activated by cytotoxic drugs were responsible for KSHV lytic cycle activation and whether their targeting could prevent such effects and further reduce PEL cell survival.

## 2. Materials and Methods

### 2.1. Cell Culture

BC3 (American Type Culture Collection, Manassas, VA, USA; ATCC) and BCBL1 (kindly provided by Prof. P. Monini, National AIDS Center, Istituto Superiore di Sanità, Rome, Italy) are human B-cell lines KSHV-infected, established from patients affected by primary effusion lymphoma (PEL). Cells were cultured in RPMI 1640 (Thermo Fisher Scientific, Waltham, MA, USA; 21870) supplemented with 10% fetal bovine serum (FBS) (Corning, NY, USA; 35-079), with L-glutamine and with streptomycin (100 µg/mL) (Corning, NY, USA; 30-002) and with penicillin (100 U/mL) (Corning, NY, USA; 25-005) in 5% CO_2_ at 37 °C.

### 2.2. Cell Treatments

To induce viral replication, PEL cell lines were treated with either 12-O-tetradecanoylphorbol 13-acetate (TPA) (20 ng/mL) (Sigma Aldrich, St Louis, MO, USA; 185361) and sodium butyrate (B) (0.3 mM) (Sigma Aldrich, St Louis, MO, USA; B5887) (TB), or with bortezomib (BZ) (20 nM) (Santa Cruz Biotechnology, Dallas, TX, USA; sc-217785) at the indicated times. BC3 and BCBL1 cell lines were treated with metformin (MET) (20 mM) (Sigma Aldrich, St Louis, MO, USA; D150659) or quercetin (Q) (50 µM) (Sigma Aldrich, St Louis, MO, USA; Q4951) at the indicated times [5,16]. To evaluate the role of HSF1 and NRF2 during KSHV replication induced by TB or BZ, a time-course assay (from 5 to 10 µM) was performed with HSF1 inhibitor, KRIBB11 (HSF1 Inhibitor, I-HSF1), (Merck, Milano, Italy. 385570) or with NRF2 inhibitor, Brusatol (HSF1 Inhibitor, I-NRF2) (from 5 to 10 nM) (Sigma Aldrich, St Louis, MO, USA; 1868) for the indicated time. In some experiments, BC3 and BCBL1 were pre-treated with I-HSF1 (5 µM), I-NRF2 (5 nM) and/or with N-acetylcysteine (NAC) (5 mM) (Sigma Aldrich, St Louis, MO, USA; A7250), for 20 min and then cultured with TPA (20 ng/mL)/sodium butyrate (0.3 mM) for the indicated time. In some experiments, BC3 cells were treated with TB in the presence of H_2_O_2_ (100 µM) for the indicated time.

In order to investigate autophagy, cells were treated with TPA (20 ng/mL)/sodium butyrate (0.3 mM) in the presence or absence of Bafilomycin A1 (BAF) (20 nM) (Santa Cruz Biotechnology Inc., Dallas, TX, USA; sc-201550), an inhibitor of vacuolar-H^+^-ATPase, for the final 4 h.

### 2.3. Cell Assay Viability

BC3 and BCBL1 cell lines were plated in 12-well plates at a density of 8 × 10^5^ cells/well. Cells were pre-treated with I-HSF1 (5 µM), I-NRF2 (5 nM) or I-HSP70 (10 µM) and then cultured with either TPA (20 ng/mL) and sodium butyrate (0.3 mM) (TB) or bortezomib (BZ) (20 nM) for 24 h in the presence or absence of NAC or H_2_O_2_.

The BC3 cell line was transiently transfected with a specific RNA duplex to knock-down NRF2 for 48 h and then treated with TPA (20 ng/mL) and sodium butyrate (0.2 mM) (TB) or bortezomib (BZ) (20 nM) for the last 24 h.

A trypan blue (Sigma Aldrich, St Louis, MO, USA; 72571) exclusion assay was performed to test cell viability. Live cells were counted by light microscopy using a Neubauer hemocytometer.

### 2.4. Antibodies

In western blotting, we used the following primary antibodies: mouse monoclonal anti-K-bZIP (1:300) (Santa Cruz Biotechnology Inc., Heidelberg, Germany; sc-69797), mouse monoclonal anti-gp64 (1:100) (Santa Cruz Biotechnology Inc., Heidelberg, Germany; sc-65444), mouse monoclonal anti-SQSTM1 (1:500) (BD Transduction Laboratories, San Jose, CA, USA; cat. no. 610833), rabbit polyclonal anti-Microtubule-Associated protein 1 light chain 3 (LC3) (1:1000) (Novus Biologicals, Cambridge, UK; NB100-2220SS), mouse monoclonal anti-HSP70 (1:100) (Santa Cruz Biotechnology Inc., Heidelberg, Germany; sc-66049), mouse monoclonal anti-NRF2 (1:100) (Santa Cruz Biotechnology Inc., Heidelberg, Germany; sc-365949), rabbit polyclonal anti-p21 (1:500) (Santa Cruz Biotechnology Inc., Heidelberg, Germany; sc-397). Mouse monoclonal anti-β-actin (1:10000) (Sigma Aldrich, St Louis, MO, USA; A5441) (1:10000) was used as the loading control. The goat polyclonal anti-mouse IgG-horseradish peroxidase (Santa Cruz Biotechnology Inc., Heidelberg, Germany; sc-2005) and anti-rabbit IgG-HRP (Santa Cruz Biotechnology Inc., Heidelberg, Germany; sc-2004) were used as secondary antibodies. All the primary and secondary antibodies were diluted in PBS-0.1% Tween20 solution containing 3% of BSA (SERVA, Reno, NV, USA; 11943.03).

In the immunofluorescence assay, mouse monoclonal anti-K-bZIP (1:300) (Santa Cruz Biotechnology Inc., Heidelberg, Germany; sc-69797) or mouse monoclonal anti-gp64 (1:100) (Santa Cruz Biotechnology Inc., Heidelberg, Germany; sc-65444) was diluted in 1XPBS and used to evaluate the KSHV lytic cycle.

### 2.5. Western Blot Analysis

1 × 10^6^ PEL cells were washed twice with 1X PBS and centrifuged at 1500 rpm for 5 min. Cells were lysed in a 1X RIPA buffer containing 150 mM Nacl, 1% NP-40 (Sigma Aldrich, NP40S), 50 mM Tris-HCl, pH 8, 0.5% deoxycholic acid (Sigma Aldrich, D6750), 0.1% SDS (Sigma Aldrich, 71736), protease (Sigma Aldrich, St Louis, MO, USA; S8830) and phosphatase inhibitors (Sodium Orthovanadate; Sigma Aldrich, St Louis, MO, USA; S6508) (Sodium Fluoride; Sigma Aldrich, St Louis., MO, USA; S7920). Following this, 10 µg of protein lysates were subjected to electrophoresis on 10% or 15% acrylamide gels. Gels were transferred to nitrocellulose membranes (Bio-Rad, 162-0115) for 2 h in Tris-glycine buffer. Membranes were blocked in PBS-0.1% Tween 20 solution containing 3% BSA, probed with specific antibodies and developed using ECL Blotting Substrate (Advansta, K-12045-D20).

### 2.6. Indirect Immunofluorescence Assay (IFA)

For immunofluorescence, BC3 and BCBL1 cell lines were treated either with TPA (20 ng/mL) and sodium butyrate (0.3 mM) or with bortezomib (20 nM) for 24 h with and without previous RNA interference to knock-down HSF1, NRF2 and SQSTM1 for 48 h. A total of 1 × 10^6^ cells were centrifuged, washed in cold 1X PBS, seeded on glass slides and air dried. Cells were fixed in 2% paraformaldehyde for 30 min and then made permeable with 0.2% Trinton X-100 (Sigma Aldrich, St Louis, MO, USA; T-8787) for 5 min at room temperature. Slides were incubated with mouse monoclonal anti-K-bZIP (1:300 in 1X PBS) (Santa Cruz Biotechnology Inc., Heidelberg, Germany; sc-69797), mouse monoclonal anti-gp64 (1:100) (Santa Cruz Biotechnology Inc., Heidelberg, Germany; sc-65444) or mouse monoclonal anti-p62/SQSTM1 (1:500 in 1X PBS) (BD Transduction Laboratories, San Jose, CA, USA; cat. no. 610833) antibody and washed three times in 1X PBS after 1 h. They were then incubated with polyclonal conjugated-Cy3 sheep anti-mouse antibody (Jackson ImmunoResearch, Cambridgeshire, UK; 515-165-062) (1:1000 in 1X PBS) for 30 min at room temperature and washed twice with 1X PBS. Cells were then incubated with 1 µg/mL of 4′,6′-diamidino-2-phenylindole (DAPI) to stain nuclei and coverslips were mounted face down using a PBS-glycerol (1:1) solution. The immunofluorescence was analyzed using a Fluorescence microscope (Zeiss) equipped with an AxioCam MRM Rev.3 at ×40 magnification.

### 2.7. Endogenous Reactive Oxygen Species (ROS) Detection

To detect reactive oxygen species, BC3 and BCBL1 cells were treated with TPA (20 ng/mL) and sodium butyrate (0.3 mM) (TB), or with bortezomib (BZ) (20 nM) for 24 h. In some experiments, PEL cell lines were pre-treated with I-HSF1 (5 µM) and I-NRF2 (5 mM) and then cultured with TPA (T) (20 ng/mL)/ sodium butyrate (B) (0.3 mM) or bortezomib (BZ) (20 nM), as described above.

Cells were stained with 2′,7′-dichlorofluorescein diacetate (DCFDA) (Thermo Fisher Scientific, Waltham, MA, USA; D399), a fluorogenic dye which diffuses into the cell. DCFDA is oxidized by ROS into 2′,7′-dichlorofluorescein, a fluorescent compound which can be detected by fluorescence spectroscopy. Cells were twice washed 1X PBS and then incubated at 37 °C with 10 µM DCFDA for 15 min. PEL cells were analyzed in FL-1 by a FACScalibur flow cytometer (BD, USA). For each analysis 10,000 events were recorded [17,18,19].

### 2.8. Transfection and Plasmids

The BC3 cell line was transiently transfected with empty vector (EV) or pDest-mCherry-EGFP-SQSTM1 (pSQSTM1) (kindly provided by Terje Johansen) [20] plasmid, using Lipofectamine 2000 as indicated by manufacture’s instructions. Briefly, 5 × 10^5^ cell were seeded into 12-wells plate in RPMI supplemented with 10% FBS and L-glutamine without antibiotics for 24 h. Then, 1 µg plasmid DNA and 3 µL Lipofectamine 2000/well were diluted in Opti-MEM medium. The mixture was added to cells for 48 h. Cells were treated with TPA (20 ng/mL) (T) and sodium butyrate (B) (0.3 mM) for the last 24 h. To evaluate the KSHV lytic activation, K-bZIP expression was assessed by western blotting assay.

### 2.9. HSF1, NRF2 and P62/SQSTM1 Knockdown by Small Interfering RNA (siRNA)

*HSF1*, *NRF2* and *SQSTM1* knockdown was performed in PEL cell lines using specific small interfering RNA. Subsequently, 3 × 10^5^ cells were seeded in 12-wells culture plate in RPMI medium supplemented with 10% fetal bovine serum (FBS) (Corning, NY, USA; 35-079), with L-glutamine and without antibiotics. Subsequently, 30 pmoli of siRNA duplex (siRNA*HSF1*, siRNA*NRF2* and siRNA*SQSTM1*) (Santa Cruz Biotechnology Inc., Dallas, TX, USA; sc-35611, sc-37030 and sc-29679) and 7 µL of Lipofectamine 2000 Transfection Reagent (Life Technologies, 11668027) were diluted in Optimem medium (Thermo Fisher Scientific, Waltham, MA, USA; 31985062) for 20 min at room temperature. The mixture was added to the cell culture for 48 h. After this, the cells were treated either with TPA (20 ng/mL) (T) and sodium butyrate (0.3 mM), or with bortezomib (20 nM) for the last 24 h.

Transfection efficiency was evaluated by Fluorescein Conjugate-A siRNA (Santa Cruz Biotechnology Inc., Dallas, TX, USA; sc-36869). A control siRNA-A (siRNA*SC*) (Santa Cruz Biotechnology Inc., Dallas, TX, USA; sc-37007) was also used [21].

### 2.10. RNA Extraction

BCBL1 cell line was treated either with TPA (20 ng/mL) and sodium butyrate (0.3 mM), or with bortezomib (20 nM) for 6 h and RNA was extracted [22]. Briefly, cells were centrifuged and washed twice in cold 1X PBS. Then, cells were lysed using 1 mL TRIzol reagent (Thermo Fisher Scientific, Waltham, MA, USA; 15596026) for 5 min at room temperature. Following this, 0.2 mL chloroform was added to the solution. To isolate total RNA, the mixture was centrifuged and 0.5mL isopropanol was added to the colorless upper aqueous phase. Extracted RNA was washed twice in 75% ethanol and then re-suspended in warmed RNAase- and DNAse-free water. To remove contaminating genomic DNA, 5 µg RNA was incubated with DNase I, according to the manufacturer’s instructions (Sigma Aldrich, St Louis, MO, USA; 11284932001). RNA samples were collected and stored at −80 °C.

### 2.11. Reverse-Transcription Quantitative PCR (qRT-PCR) and Quantitative PCR (qPCR)

1 µg total RNA was used to synthesize single-stranded cDNA, using SuperScript III Reverse Transcriptase Kit (Thermo Fisher Scientific, Waltham, MA, USA; 18064014). A total of 2 µL/well (10 ng/sample) of template was mixed to SYBR Master (Applied Biosystems, Foster City, CA, USA; 4472908), RNAase-/DNAse-free water and primers in 20 µL volume.

To perform qRT-PCR, the following conditions were used: 50 °C for 2 min, 95 °C for 2 min, 95 °C for 15 s and 60 °C for 1 min (40 cycles). The following primers were used:

SQSTM1 (FW GGAGCCAGAGAACAAGTACC; RW CTCGCTCTTTCAGTTTCATGTTC)

ACTA(FWTCACCCACACTGTGCCATCCTACGA;RWCAGCGGAACCGCTCATTGCCAATGG) [23,24].

Target mRNA level was normalized to housekeeping mRNA actin and analyzed, comparing treated (TB or BZ) to untreated samples.

To evaluate KSHV viral production, the BC3 cell line was pre-treated with I-HSF1 (5 µM) for 1 h and then with NAC (5 mM) for the last 30 min. Cells were treated with TPA (20 ng/mL) (T)/Butyrate (B) (0.3 mM) for 24 h. Supernatants were collected and qRT-PCR was performed to assess KSHV DNA using the HHV-8 Elite MGB kit (ELITechGroup, Puteaux, France; RTS038PLD).

### 2.12. Densitometric Analysis

The quantification of proteins bands was performed by densitometric analysis using the Image J software, which was downloaded from the NIH web site (available online: http://imagej.nih.gov).

### 2.13. Statistical Analysis

Results are represented by the mean ± standard deviation (SD) of at least three independent experiments. Differences were considered statistically significant for *p*-value <0.05.

## 3. Results

### 3.1. TPA/Butyrate (TB) and Bortezomib (BZ) Increase Intracellular ROS and Promote KSHV Lytic Antigen Expression, while Quercetin (Q) and Metformin (MET) Reduce ROS and Do Not Induce This Effect in PEL Cells

**Figure 1:**
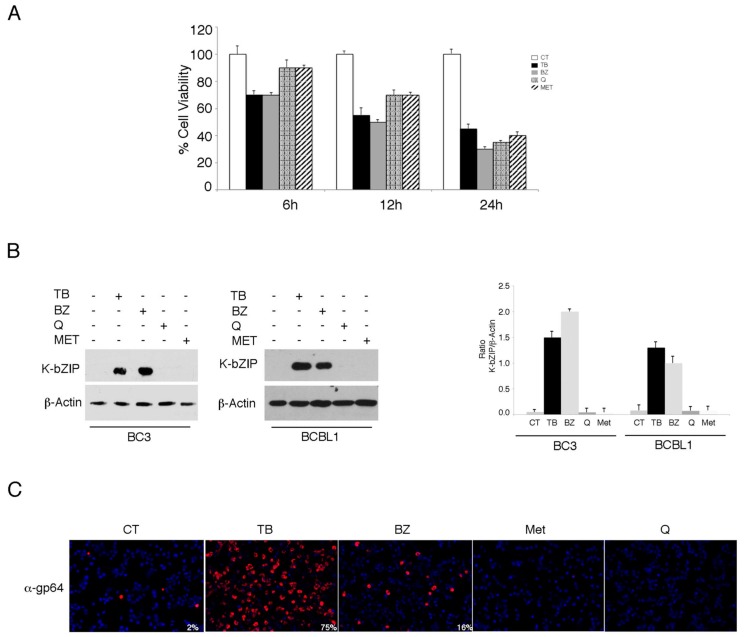
12-O-tetradecanoylphorbol 13-acetate (TPA) in combination with sodium butyrate (TB) and bortezomib (BZ) induce the Kaposi sarcoma-associated herpesvirus (KSHV) lytic cycle, while metformin and quercetin fail to do so in PEL cells. (**A**) The BC3 cell line was treated with either TPA (T) (20 ng/mL) and sodium butyrate (0.3 mM) in combination (TB), bortezomib (BZ) (20 nM), metformin (MET) (20 mM), or quercetin (Q) (15 µM). Cell viability was evaluated by trypan blue exclusion in a time course assay. Histograms represent the mean ± standard deviation (SD) of at least three independent experiments. (**B**) BC3 and BCBL1 cell lines were treated with TPA (T) (20 ng/mL) and sodium butyrate (B) (0.3 mM) in combination (TB), bortezomib (BZ) (20 nM), metformin (MET) (20 mM), or quercetin (Q) (15 µM) for 24 h. K-bZIP and β-Actin expression was evaluated by western blotting. Densitometric analysis was performed using Image J software and the ratio of K-bZIP versus β-Actin was calculated. Histograms represent the mean ± standard deviation (SD) of at least three independent experiments. (**C**) The BC3 cell line was treated with TPA (T) (20 ng/mL) and sodium butyrate (B) (0.3 mM) in combination (TB), bortezomib (BZ) (20 nM), metformin (MET) (20 mM), or quercetin (Q) (15 µM) for 24 h, and was analyzed by IFA for the expression of the late lytic antigen gp64. The percentage of gp64-positive cells is also indicated. Magnification 20×.

**Figure 2:**
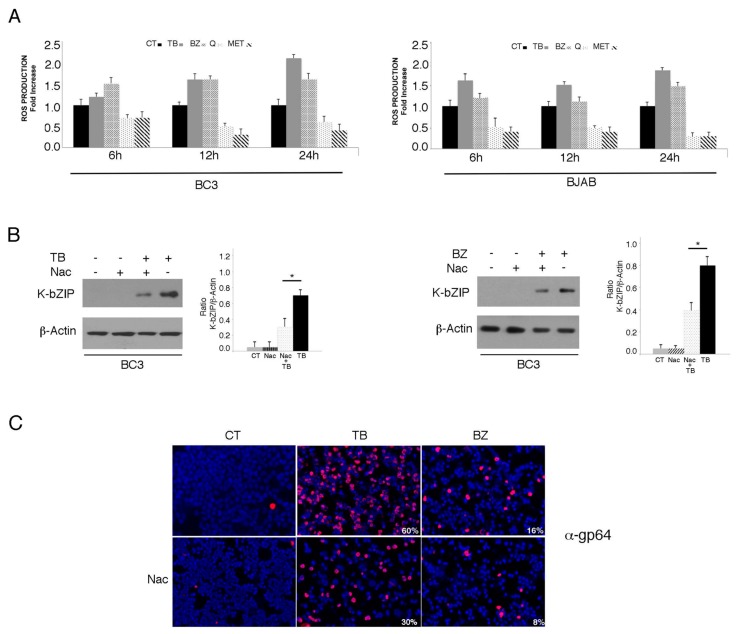
Oxygen reactive species (ROS) levels increase with TB- and BZ-treated PEL cells, and decrease in MET- and Q-treated PEL cells. (**A**) KSHV-infected BC3 or uninfected BJAB cell lines were cultured with TB (20 ng/mL and 0.3 mM), BZ (20 nM), MET (5 mM) or Q (15 µM) (6, 12 and 24 h) and ROS production was assessed by FACS analysis in a time-dependent manner using DCFDA detection assay. Histograms represent the fold increase of treated versus untreated cells. Standard deviations (SD) are shown. (**B**) KSHV lytic antigen K-bZIP expression in TB- (20 ng/mL and 0.3 mM) or BZ- (20 nM) treated cells, in the presence or in the absence of N-Acetyl cysteine (Nac) (5 mM), assessed by western blotting analysis. β-Actin was used as a loading control. Densitometric analysis has been performed using Image J software and the ratio of K-bZIP versus β-Actin was calculated. Histograms represent the mean ± standard deviation (SD) of at least three independent experiments. p* value < 0.05. (**C**) Late lytic antigen gp64 expression was evaluated by IFA in TB- or BZ-treated PEL cells, in the presence or in the absence of NAC. The percentage of gp64-positive cells is also indicated. Magnification 20×.

We investigated the impact of four different cytotoxic treatments, namely TPA in combination with sodium butyrate (TB), bortezomib (BZ), quercetin (Q) and metformin (MET), on the activation of the KSHV lytic cycle in PEL cells. These drugs, able to induce apoptosis in these cells [3,4,5,25] with similar kinetics (Figure 1A), were used at concentrations that inhibit 50% of cell survival. As shown in Figure 1B,C, both TB and BZ induced the expression of early and late viral lytic antigens K-bZIP and gp64, respectively, as indicated by western blot or by IFA, while Q and MET failed do so. Searching for the mechanisms that could underlie these differing impacts on the lytic cycle activation, we found that TB and BZ increased ROS while MET and Q reduced them, as evaluated by FACS analysis using DCFDA staining (Figure 2A). Similarly, TB and BZ increased ROS while MET and Q decreased them in BJAB, a B lymphoma cell line that does not harbor the viral genome (Figure 2A), suggesting that ROS modulation was independent of KSHV infection. The reduction of K-bZIP expression by the ROS scavenger N-acetylcysteine (NAC) (Figure 2B), and the decreased number of cells expressing the KSHV late lytic antigen gp64 (Figure 2C), as evaluated by IFA, indicated that the ROS increase played a role in the activation of KSHV lytic cycle in PEL cells undergoing TB or BZ treatment.

### 3.2. p62/SQSTM1 is Up-Regulated by TB and BZ and Down-Regulated By MET And Q

**Figure 3:**
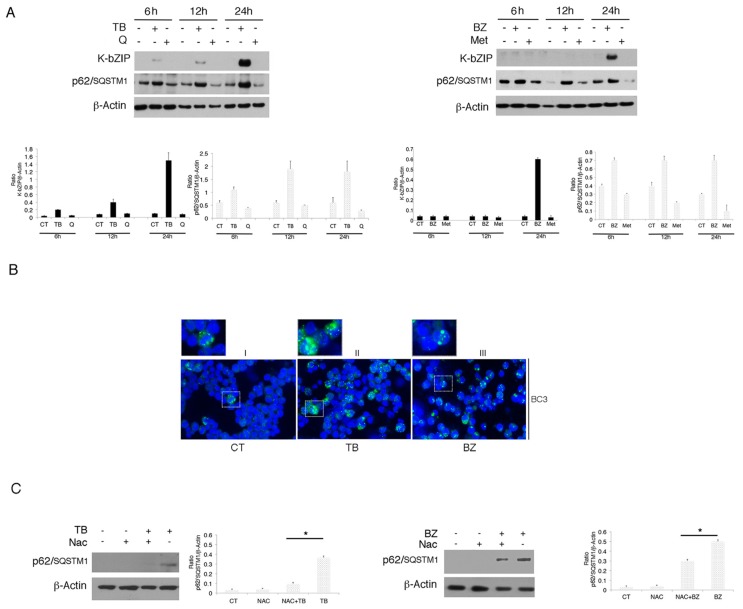
p62/SQSTM1 protein accumulates with TB and BZ treatments in the BC3 cell line. (**A**) p62/SQSTM1 expression was evaluated by western blotting in a time-dependent manner in PEL cells treated with TB (20 ng/mL) (0.3 mM) or BZ (20 nM) in the BC3 cell line. K-bZIP and β-Actin were used as KSHV lytic infection markers and a loading control, respectively. Densitometric analysis was performed using Image J software and the K-bZIP or SQSTM1/β-Actin ratio was calculated. Histograms represent the mean ± standard deviation (SD) of three independent experiments. (**B**) p62/SQSTM1 accumulated in the BC3 cell line treated with TB or BZ, as indicated by IFA experiments performed after 24 h of treatment (panel II and III, green). DAPI was used for nuclei staining (blue). Magnification ×40. The dashed rectangular box displays a zoomed-view of SQSTM1-positive cells. (**C**) p62/SQTM1 expression was evaluated in TB- and BZ- treated cells in the presence or absence of NAC. Densitometric analysis was performed using Image J software and the ratio of K-bZIP or SQSTM1 versus β-Actin was calculated. Histograms represent the mean ± standard deviation (SD) of at least three independent experiments. * *p*-value < 0.05.

It has been reported that oxidative stress may lead to the up-regulation of p62/SQSTM1 expression [26]. Thus, we investigated if its expression could be up-regulated by TB or BZ treatment, to help cells cope with oxidative stress. As shown in Figure 3A, we found that p62/SQSTM1 accumulated after 6, 12 and 24 h of TB or BZ treatments, the same time points in which these drugs increased ROS and activated KSHV lytic antigen expression. Conversely, Q and MET, which reduced ROS and down-regulated p62/SQSTM1 expression, did not induce viral replication. The accumulation of p62/SQSTM1 in TB- and BZ-treated PEL cells was then demonstrated by IFA experiments that also evidenced its different intracellular localization in cells following TB or BZ treatment (Figure 3B). This could be due to the BZ-mediated activation of kinases phosphorylating p62/SQSTM1 and leading to the formation of larger aggresomes. Subsequently, we correlated p62/SQSTM1 up-regulation with ROS increase. The finding that p62/SQSTM1 expression decreased in the presence of the ROS scavenger NAC in TB- and BZ-treated cells indicated that oxidative stress played a role in increasing its expression (Figure 3C).

### 3.3. p62/SQSTM1 Up-Regulation is not Dependent on Autophagy Reduction at 6 and 12 h, while Autophagy Inhibition Contributes to Its Accumulation at 24 h after TB or BZ Treatments

**Figure 4:**
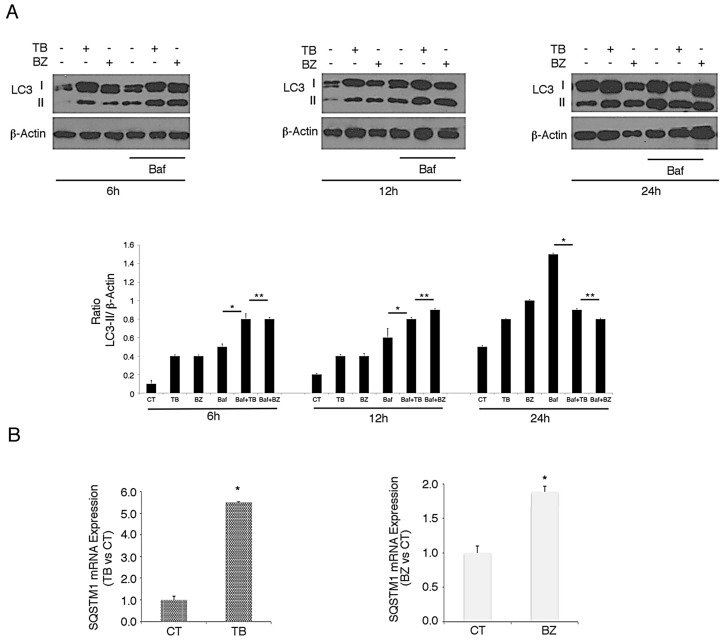
TB and BZ, which activate the KSHV lytic cycle and induce autophagy, increase SQSTM1 RNA in PEL cells. BC3 PEL cells were treated with (**A**) TB or (**B**) BZ in the presence or absence of bafilomycin (BAF) (20 nM) for the final 3 h, and LC3-I/II was evaluated by western blotting analysis. β-Actin was used as a loading control. Densitometric analysis was performed using Image J software and the LC3-II/β-Actin ratio was calculated. Histograms represent the mean ±standard deviation (SD) of at least three independent experiments. * *p*-value < 0.05, ** *p*-value < 0.05. (**B**) BC3 cell line was treated with TB or BZ for 6 h and *SQSTM1* RNA was evaluated by qRT-PCR. Target mRNA level was normalized to actin gene and analyzed to compare treated (TB or BZ) with untreated samples. Data are plotted in histograms showing standard deviation (SD). * *p*-value < 0.05.

As p62/SQSTM1 is a protein mainly degraded through autophagy [11], we evaluated whether p62/SQSTM1 accumulation in TB- and BZ-treated PEL cells could be due to the inhibition of autophagic flux. For this aim, we evaluated LC3-I/II expression in PEL cells treated with TB or BZ after 6–12 or 24 h of treatment, in the presence or absence of bafilomycin (BAF). Bafilomycin is an inhibitor of vacuolar H^+^ ATPase (V-ATPase) that inhibits the last autophagic steps and thus LC3-II degradation, allowing us to better evaluate its formation. As shown in Figure 4A, after 6 and 12 h, LC3-II expression increased in cells treated with TB or BZ in the presence of BAF, indicating that the autophagic flux was complete and that p62 accumulation did not depend on the reduction of autophagy at these time points. Similar to our previous findings [27], after 24 h of TB or BZ treatment LC3-II did not further accumulate in the presence of BAF in comparison to control cells (see Figure 4A), indicating that autophagy was blocked in the final stages and contributed to p62/SQSTM1 accumulation. We then investigated whether the p62/SQSTM1 up-regulation observed after 6 h of TB or BZ treatment was dependent on mRNA increase. Towards this aim, we performed qRT-PCR analysis and, as shown in in Figure 4B, we found that SQSTM1 mRNA increased in cells undergoing TB or BZ treatment. These results suggest that an increase of transcription and/or changes in mRNA stability and degradation could be leading to the up-regulation of p62/SQSTM1 observed at the time in which the autophagic flux was complete.

### 3.4. p62/SQSTM1 Plays a Role in KSHV Lytic Antigen Expression Induced by TB or BZ

**Figure 5:**
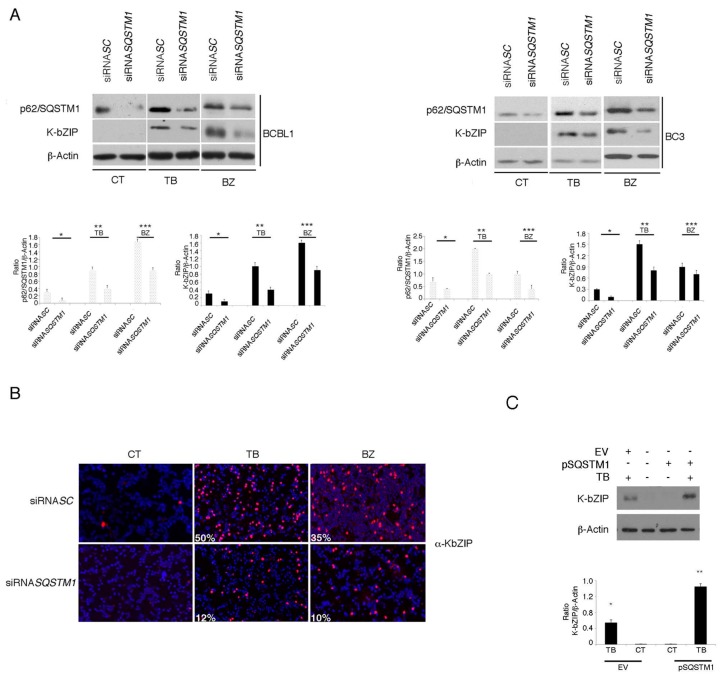
SQSTM1 RNA interference reduces K-bZIP expression in PEL cell lines. (**A**) p62/SQSTM1 expression following RNA interference using a specific siRNA*SQSTM1*, before treating PEL cells with TB or BZ. Both p62/SQSTM1 and K-bZIP expression were evaluated by western blotting, and siRNA*SC* was used as a control. Densitometric analysis was performed using Image J software and the ratio of p62/SQSTM1 and K-bZIP versus β-Actin was calculated. Histograms represent the mean ± standard deviation (SD) of three independent experiments. p* < 0.05 (siRNA *SQSTM1* vs siRNA *SC*), p** < 0.05 (siRNA *SQSTM1* vs siRNA *SC*; TB-treated) and p*** < 0.05 (siRNA *SQSTM1* vs siRNA *SC*; BZ-treated) (**B**). K-bZIP expression (red staining) was evaluated by immunofluorescence assay in *SQSTM1* knocked-down BCBL1 cells. The percentage of K-bZIP-positive cells is indicated. DAPI was used to stain nuclei (blue). Images are 40× magnification. All results are representative of three independent experiments. (**C**) K-bZIP expression in TB-treated BC3 cells overexpressing SQSTM1, as evaluated by western blot analysis. Densitometric analysis was performed using Image J software and the ratio of p62/SQSTM1 and K-bZIP versus β-Actin was calculated. Histograms represent the mean ± standard deviation (SD) of three independent experiments. p* < 0.05 (TB vs CT; EV-transfection)and p** < 0.05 (TB vs CT; pSQSTM1-transfection).

As p62/SQSTM1 expression increased in PEL cells undergoing TB or BZ, the same treatments that induced KSHV lytic cycle, the role of p62/SQSTM1 in this process was evaluated. We performed *SQSTM1* knocking-down by using specific siRNA and found that it led to a reduction of K-bZIP expression in PEL cells treated with TB or BZ (Figure 5A). These results were confirmed by immunofluorescence experiments that showed a reduction of K-bZIP-positive cells after *SQSTM1* silencing (Figure 5B), further indicating that p62/SQSTM1 plays a role in KSHV lytic antigen expression induced by TB or BZ in PEL cells. To confirm the importance of p62/SQSTM1 in supporting the KSHV lytic cycle, we also overexpressed this molecule by using a plasmid expression vector and, as shown in Figure 5C, p62/SQSTM1 overexpression caused increased K-bZIP expression in TB-treated cells.

### 3.5. The Main Mediators of Cell Stress Responses, HSF1 and NRF2, Promote P62/SQSTM1 And KSHV Lytic Antigen Expression in TB- or BZ-Treated PEL Cells

**Figure 6:**
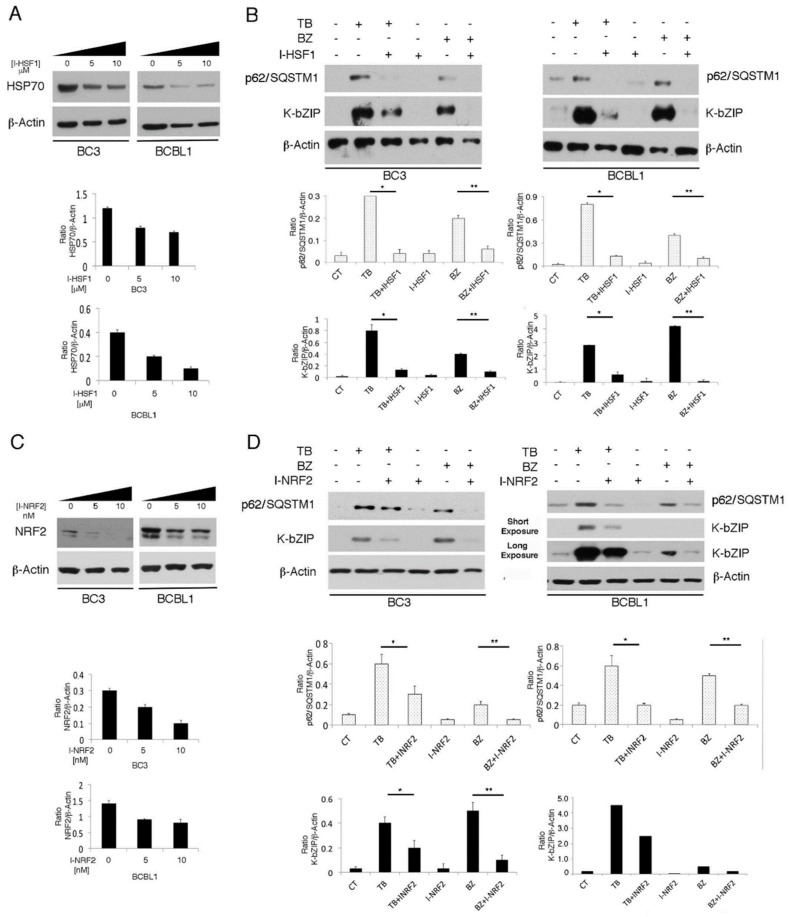
HSF1 and NRF2 inhibition counteracts the KSHV lytic cycle induced by TB and BZ in PEL cells. (**A**) and (**C**): HSF1 and NRF2 activity was inhibited in a time-course assay by I-HSF1 (KRIBB11) or I-NRF2 (brusatol), as revealed by HSP70 or NRF2 expression evaluated by western blotting analysis. β-Actin was used as a loading control. Densitometric analysis was performed using Image J software and the ratio of HSP70 or NRF2 versus β-Actin was calculated. Histograms represent the mean ± standard deviation (SD) of three independent experiments. (**B)** and (**D**): K-bZIP lytic antigen and p62/SQSTM1 expression in TB- or BZ-treated PEL cells in the presence or absence of HSF1 (5 µM) or NRF2 (5 nM) inhibitors (I-HSF1 and I-NRF2) was analyzed by western blotting after 24 h of treatment. Densitometric analysis was performed using Image J software and the ratio of K-bZIP or p62/SQSTM1 versus β-Actin was calculated. Histograms represent the mean ± standard deviation (SD) of three independent experiments. p* < 0.05 (TB/I-HSF1 or I-NRF2 vs TB), p** < 0.05 (BZ/I-HSF1 or I-NRF2 vs BZ).

**Figure 7:**
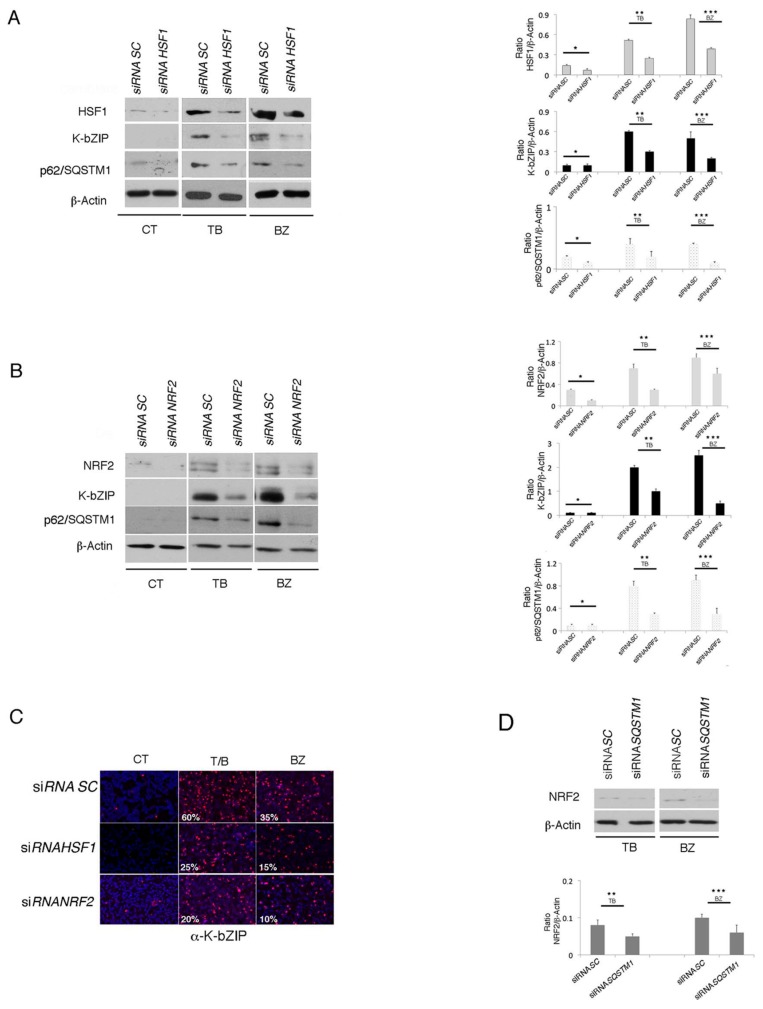
HSF1 and NRF2 RNA interference reduces K-bZIP expression in TB- or BZ-treated PEL cells. (**A**) HSF1 and (**B**) NRF2 knocking-down by specific siRNA decreased K-bZIP and p62/SQSTM1 expression in BCBL1 treated with TB or BZ. Densitometric analysis was performed using Image J software and the ratio of K-bZIP, HSF1, NRF2 or SQSTM1 versus β-Actin was calculated. Histograms represent the mean ± standard deviation (SD) of at least three independent experiments. p* < 0.05 (siRNA *HSF1* or siRNA *NRF2* vs siRNA *SC*), p** < 0.05 (siRNA *HSF1* or siRNA *NRF2* vs siRNA *SC*, TB-treated) and p*** < 0.05 (siRNA *HSF1* or siRNA *NRF2* vs siRNA *SC*, BZ-treated). (**C**) Immunofluorescence analysis of K-bZIP expression (red) in HSF1 and NRF2 knocked-down cells treated for 24 h with TB or BZ. DAPI was used to stain nuclei (blue). Percentage of K-bZIP-positive cells is also indicated. Objective magnification 20×. (**D**) NRF2 expression in *SQSTM1* knocked-down cells, induced to lytic replication by TB or BZ, as evaluated by western blotting. Densitometric analysis was performed using Image J software and the ratio of NRF2 versus β-Actin was calculated. Histograms represent the mean ± standard deviation (SD) of three independent experiments. p** < 0.05 (siRNA *SQSTM1* vs siRNA *SC*, TB-treated), and p*** < 0.05 (siRNA *SQSTM1* vs siRNA *SC*, BZ-treated).

p62/SQSTM1 is a common transcription target of HSF1 and NRF2, activated during the main mammalian cellular responses to stress (the heat shock and anti-oxidant responses, respectively) [9]. In order to investigate the role HSF1 and NRF2 in p62/SQSTM1 up-regulation and the activation of KSHV lytic antigen expression, we pharmacologically inhibited these transcription factors. In a dose response assay, we found that the HSF1 inhibitor KRIBB11 (I-HSF1) used at 5 µM was able to reduce HSF1 activity, as revealed by the decreased expression of its target heat shock protein (HSP) 70 (Figure 6A), and reduced p62/SQSTM1 and K-bZIP expression in TB- or BZ-treated PEL cell lines (Figure 6B). Furthermore, the inhibition of NRF2 by brusatol (I-NRF2) at concentrations able to reduce NRF2 expression (5 nM) (Figure 6C), decreased p62/SQSTM1 and K-bZIP expression (Figure 6D), suggesting that both HSF1 and NRF2 transcription factors played a role in the KSHV lytic antigen expression induced by TB or BZ in PEL cells. Given that pharmacological inhibitors could have off-target effects, to better elucidate the role of these transcription factors in KSHV lytic antigen expression and p62/SQSTM1 up-regulation, we performed HSF1 or NRF2 silencing by using specific siRNA. As shown in Figure 7A,B, HSF1 and NRF2 knocking-down reduced both p62/SQSTM1 and K-bZIP expression in TB- and BZ-treated cells. The results confirmed the reduction of the percentage of K-bZIP-positive cells in NRF2 and HSF1 silenced cells undergoing TB or BZ treatment (Figure 7C). Considered together, these results indicate that the main mediators of cellular responses to stress, HSF1 and NRF2, and their target p62/SQSTM1, played a role in the induction of KSHV lytic antigen expression induced by TB or BZ in PEL cells. Notably, p62/SQSTM1 siRNA reduced NRF2 expression (Figure 7D), in agreement with previous studies indicating that a positive feedback loop between the two molecules may occur [28,29,30].

### 3.6. The Inhibition of HSF1, NRF2 and p62/SQSTM1 (siRNA) Further Increases ROS and Cytotoxicity Induced by TB or BZ in PEL Cells

**Figure 8:**
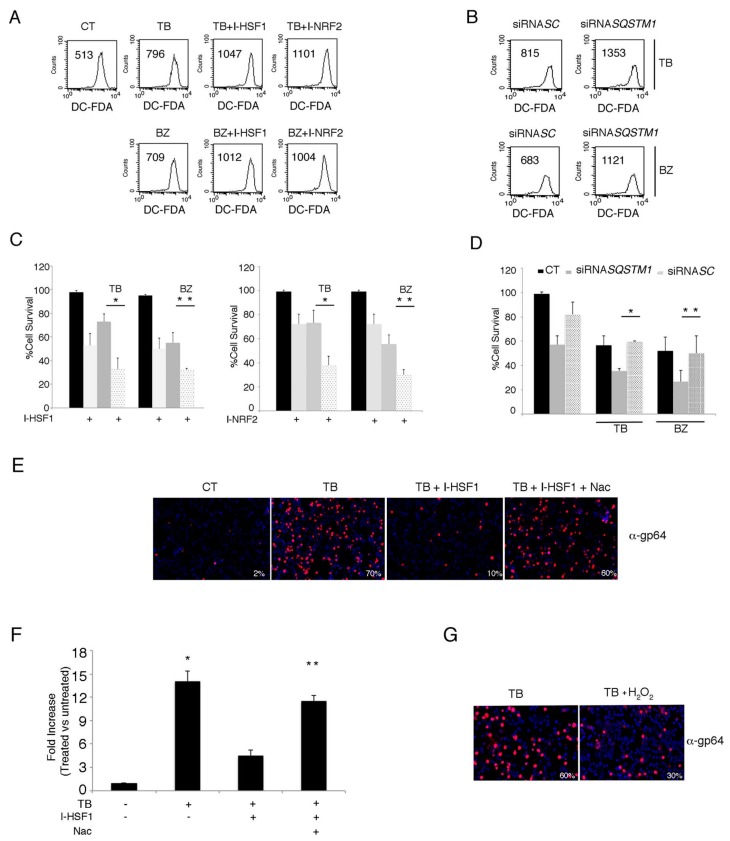
HSF1, NRF2 and SQSTM1 inhibition increases endogenous ROS and decreases PEL cell viability in TB- and BZ-treated PEL cells. (**A**) Intracellular ROS in the BC3 cell line treated with or without HSF1 and NRF2 inhibitors in the presence of TB or BZ. Flow-cytometric analysis was performed to measure ROS using DCFDA staining after 6 h of treatment. The mean of fluorescence intensity is indicated and one representative experiment out of three is shown. (**B**) Intracellular ROS in BC3 cells *SQSTM1* knocked-down during treatment with TB or BZ for 6 h. (**C**) Cell viability as evaluated by trypan blue exclusion assay in BC3 cell line treated with TB or BZ, with or without I-HSF1 or I-NRF2 inhibitors for 24 h. Data are plotted as histograms showing mean ± standard deviation (SD). p* < 0.05, p** < 0.05. (**D**) NRF2 expression in p62/*SQSTM1* knocked-down PEL cells. siRNASC was used as a control. p* < 0.05, p** < 0.05. (**E**) gp64 expression of PEL cells treated with TB alone or in combination with HSF1 inhibitor in the presence or absence of NAC. Magnification 20×. (**F**) KSHV release in the supernatants of BC3 cells treated with TB in the presence or absence HSF1 inhibitor and in the presence or absence of NAC. Histograms represent the KSHV DNA fold increase of treated to untreated control cells. The mean ± standard deviation (SD) calculated by statistical software is shown. p* < 0.05, p** < 0.05. (**G**) gp64 expression of PEL cells treated with TB in the presence or absence of H_2_O_2_. Magnification 20×.

Since HSF1, NRF2 and p62/SQSTM1 proteins may be activated in the course of oxidative stress, we evaluated the level of intracellular ROS in PEL cells treated with TB or BZ in the presence or absence of HSF1 or NRF2 inhibitors KRIBB11 (I-HSF1) and brusatol (I-NRF2) respectively. *SQSTM1* (siRNA) was also evaluated. As shown in Figure 8A, HSF1 and NRF2 inhibitors led to a further increase of ROS level in comparison with TB or BZ single treatments, and similarly, *SQSTM1* RNA knocking-down also exerted this effect (Figure 8B), likely due to the positive feedback loop between p62 and NRF2 [28,29]. These results suggest that HSF1, NRF2 and p62/SQSTM1 are required to maintain the ROS increase at a moderate level, allowing KSHV lytic cycle activation in TB- or BZ-treated PEL cells. Indeed, when ROS level further increased by the combination of TB or BZ with *SQSTM1* silencing, HSF1 or NRF2 inhibition, the cytotoxicity increased (Figure 8C,D) and likely rendered the cellular environment unsuitable for viral replication. This hypothesis was confirmed by the findings that NAC supplementation rescued the ability of TB to activate KSHV p64 lytic antigen expression (Figure 8E) and to induce viral release (Figure 8F) in the presence of HSF1 inhibitor. Conversely, the addition of H_2_O_2_ to TB reduced KSHV late lytic expression (Figure 8G), further highlighting that the ROS level is critical for virus replication.

## 4. Discussion

The results obtained in this study indicate that cytotoxic drugs which increase ROS levels, such as TB and BZ, activate the KSHV lytic cycle, while those that decrease ROS levels, such as MET and Q, although cytotoxic for PEL cells, fail to do so. Therefore, the induction of viral reactivation from latency appears to correlate with the increase or decrease of intracellular ROS, whose role was highlighted by the use of NAC that prevented viral lytic antigen expression in TB- or BZ-treated PEL cells. Previous studies have attempted to investigate which molecular mechanisms could be responsible for lytic cycle activation by some cytotoxic treatments. It has been reported, for example, that the induction of apoptosis is neither necessary nor sufficient to induce gammaherpesvirus replication [25,31,32]. Additionally, autophagy activation, while promoting the replication of gammaherpesviruses such as KSHV and EBV, is not sufficient per se to trigger the replicative process [21,27]. Indeed, as we have previously demonstrated, quercetin (Q) and metformin (MET) are able to activate autophagy in PEL cells [4,5], similar to BZ or TB treatments [2,25].

The role of oxidative stress in activating KSHV has been shown by our laboratory and that of others [6,33]. Besides confirming the importance of ROS in reactivating KSHV from latency, this study also suggests that, in order to allow KSHV to reactivate from latency in PEL cells, ROS increases must be kept to moderate levels by HSF1 and NRF2 transcription factors and p62/SQSTM1 [9]. Their pharmacologic or genetic inhibition indeed further increased ROS in comparison to TB or BZ single treatments, likely rendering the cellular environment unsuitable for viral replication. Indeed, the targeting of HSF1 and NRF2, as well as p62/SQSTM1 silencing, enhanced the cytotoxic effect of TB or BZ treatments in PEL cells.

It is possible that different levels of ROS determine whether KSHV remains latent or switches to a lytic cycle or no longer replicates. In particular, when the ROS level is low, KSHV continues to remain in a latent state; conversely, when the ROS level slightly increases, the virus may sense the cellular stress and starts replicating before cells will possibly end up dying. Accordingly, a mild ROS increase has been shown to induce a low DNA damage that may lead to the activation of the growth arrest function of wild type p53, through the transcription of p21. Interestingly, the p53–p21 axis has been reported to be essential for KSHV replication [3,8]. However, when the level of ROS is too high, the activation of the pro-apoptotic function p53 may occur [34] and cell death may be induced [35].

**Figure 9:**
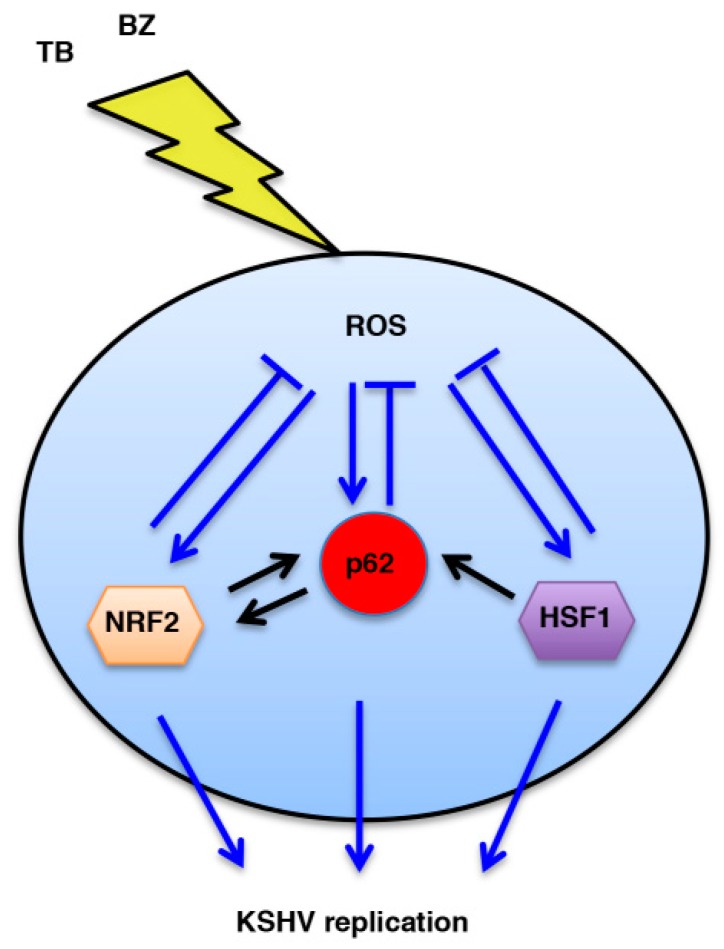
Schematic model. Interplay between HSF1, NRF2 and p62/SQSTM1 proteins and ROS intracellular production during KSHV replication induced by TB or BZ tratements.

Regarding BZ, it has been previously reported that it induces an ROS increase in lymphoma cells [36] and induces Endoplasmic Reticulum (ER) stress [37]. We have previously shown that BZ also induced ER stress in PEL cells and that through this effect it activated the autophagic process [2,25]. As stated above, ROS plays a role in activating KSHV replication during several treatments [6,33]. Interestingly, the inhibition of NRF2, the main mediator of the antioxidant response, may reactivate KSHV from latency [13]. However, based on the results obtained in this study, we observed that, in order to activate KSHV replication, the increase of ROS must be kept at a moderate level by HSF1, NRF2 and p62/SQSTM1 (Figure 9). It is a clever strategy for viruses to initiate a lytic replication in cells undergoing mild stress that could possibly increase and lead to a condition in which both cellular and virus survival is no longer guaranteed. The latter event occurs when the level of ROS is too high or, conversely, too low. Indeed, in comparison to normal cells, cancer cells are usually dependent on a higher level of ROS that promotes cell survival by maintaining activated oncogenic pathways, such as PI3K and NF-κB [38]. Accordingly, we have observed MET or Q treatments that reduced ROS and the activation of oncogenic pathways induced PEL cell death [4,5,39].

In conclusion, this study suggests that targeting ROS or the pathways that regulate the ROS level, such as NRF2, HSF1 or p62/SQSTM1, could be a promising strategy to increase PEL cell death and concomitantly restrain viral replication induced by cytotoxic drugs such as TB or BZ.

## References

[1] Lukac D.M., Yuan Y., Arvin A., Campadelli-Fiume G., Mocarski E., Moore P.S., Roizman B., Whitley R., Yamanishi K. (2007). Reactivation and lytic replication of KSHV. Human Herpesviruses: Biology, Therapy, and Immunoprophylaxis.

[2] Granato M., Santarelli R., Lotti L.V., Di Renzo L., Gonnella R., Garufi A., Trivedi P., Frati L., D’Orazi G., Faggioni A. (2013). JNK and macroautophagy activation by bortezomib has a pro-survival effect in primary effusion lymphoma cells. PLoS ONE.

[3] Gonnella R., Yadav S., Gilardini Montani M.S., Granato M., Santarelli R., Garufi A., D’Orazi G., Faggioni A., Cirone M. (2017). Oxidant species are involved in T/B-mediated ERK1/2 phosphorylation that activates p53-p21 axis to promote KSHV lytic cycle in PEL cells. Free Rad. Biol. Med..

[4] Granato M., Rizzello C., Gilardini Montani M.S., Cuomo L., Vitillo M., Santarelli R., Gonnella R., D’Orazi G., Faggioni A., Cirone M. (2017). Quercetin induces apoptosis and autophagy in primary effusion lymphoma cells by inhibiting PI3K/AKT/mTOR and STAT3 signaling pathways. J. Nutr. Biochem..

[5] Granato M., Gilardini Montani M.S., Romeo M.A., Santarelli R., Gonnella R., D’Orazi G., Faggioni A., Cirone M. (2017). Metformin triggers apoptosis in PEL cells and alters bortezomib-induced Unfolded Protein Response increasing its cytotoxicity and inhibiting KSHV lytic cycle activation. Cell Signal..

[6] Li X., Feng J., Sun R. (2011). Oxidative stress induces reactivation of Kaposi’s sarcoma-associated herpesvirus and death of primary effusion lymphoma cells. J. Virol..

[7] Cohen A., Brodie C., Sarid R. (2006). An essential role of ERK signalling in TPA-induced reactivation of Kaposi’s sarcoma-associated herpesvirus. J. Gener. Virol..

[8] Balistreri G., Viiliainen J., Turunen M., Diaz R., Lyly L., Pekkonen P., Rantala J., Ojala K., Sarek G., Teesalu M. (2016). Oncogenic Herpesvirus Utilizes Stress-Induced Cell Cycle Checkpoints for Efficient Lytic Replication. PLoS Pathog..

[9] Dayalan Naidu S., Kostov R.V., Dinkova-Kostova A.T. (2015). Transcription factors Hsf1 and Nrf2 engage in crosstalk for cytoprotection. Trends Pharmacol. Sci..

[10] Katsuragi Y., Ichimura Y., Komatsu M. (2015). p62/SQSTM1 functions as a signaling hub and an autophagy adaptor. FEBS J..

[11] Klionsky D.J., Abdelmohsen K., Abe A., Abedin M.J., Abeliovich H., Acevedo Arozena A., Adachi H., Adams C.M., Adams P.D., Adeli K. (2016). Guidelines for the use and interpretation of assays for monitoring autophagy (3rd edition). Autophagy.

[12] Metz P., Chiramel A., Chatel-Chaix L., Alvisi G., Bankhead P., Mora-Rodriguez R., Long G., Hamacher-Brady A., Brady N.R., Bartenschlager R. (2015). Dengue Virus Inhibition of Autophagic Flux and Dependency of Viral Replication on Proteasomal Degradation of the Autophagy Receptor p62. J. Virol..

[13] Gjyshi O., Roy A., Dutta S., Veettil M.V., Dutta D., Chandran B. (2015). Activated Nrf2 Interacts with Kaposi’s Sarcoma-Associated Herpesvirus Latency Protein LANA-1 and Host Protein KAP1 To Mediate Global Lytic Gene Repression. J. Virol..

[14] Baquero-Perez B., Whitehouse A. (2015). Hsp70 Isoforms Are Essential for the Formation of Kaposi’s Sarcoma-Associated Herpesvirus Replication and Transcription Compartments. PLoS Pathog..

[15] Grundhoff A., Ganem D. (2004). Inefficient establishment of KSHV latency suggests an additional role for continued lytic replication in Kaposi sarcoma pathogenesis. J. Clin. Investig..

[16] Granato M., Rizzello C., Romeo M.A., Yadav S., Santarelli R., D’Orazi G., Faggioni A., Cirone M. (2016). Concomitant reduction of c-Myc expression and PI3K/AKT/mTOR signaling by quercetin induces a strong cytotoxic effect against Burkitt’s lymphoma. Int. J. Biochem. Cell Biol..

[17] Gilardini Montani M.S., Granato M., Cuomo L., Valia S., Di Renzo L., D’Orazi G., Faggioni A., Cirone M. (2016). High glucose and hyperglycemic sera from type 2 diabetic patients impair DC differentiation by inducing ROS and activating Wnt/beta-catenin and p38 MAPK. Biochim. Biophys. Acta.

[18] Gilardini Montani M.S., Santarelli R., Granato M., Gonnella R., Torrisi M.R., Faggioni A., Cirone M. (2018). EBV reduces autophagy, intracellular ROS and mitochondria to impair monocyte survival and differentiation. Autophagy.

[19] Gilardini Montani M.S., Santarelli R., Falcinelli L., Gonnella R., Granato M., Di Renzo L., Cuomo L., Vitillo M., Faggioni A., Cirone M. (2018). EBV up-regulates PD-L1 on the surface of primary monocytes by increasing ROS and activating TLR signaling and STAT3. J. Leukoc. Biol..

[20] Bjorkoy G., Lamark T., Brech A., Outzen H., Perander M., Overvatn A., Stenmark H., Johansen T. (2005). p62/SQSTM1 forms protein aggregates degraded by autophagy and has a protective effect on huntingtin-induced cell death. J. Cell Biol..

[21] Granato M., Santarelli R., Farina A., Gonnella R., Lotti L.V., Faggioni A., Cirone M. (2014). Epstein-barr virus blocks the autophagic flux and appropriates the autophagic machinery to enhance viral replication. J. Virol..

[22] Granato M., Feederle R., Farina A., Gonnella R., Santarelli R., Hub B., Faggioni A., Delecluse H.J. (2008). Deletion of Epstein-Barr virus BFLF2 leads to impaired viral DNA packaging and primary egress as well as to the production of defective viral particles. J. Virol..

[23] Medvedev R., Ploen D., Spengler C., Elgner F., Ren H., Bunten S., Hildt E. (2017). HCV-induced oxidative stress by inhibition of Nrf2 triggers autophagy and favors release of viral particles. Free Rad. Biol. Med..

[24] Cheng F., Pekkonen P., Laurinavicius S., Sugiyama N., Henderson S., Gunther T., Rantanen V., Kaivanto E., Aavikko M., Sarek G. (2011). KSHV-initiated notch activation leads to membrane-type-1 matrix metalloproteinase-dependent lymphatic endothelial-to-mesenchymal transition. Cell Host Microbe.

[25] Granato M., Romeo M.A., Tiano M.S., Santarelli R., Gonnella R., Gilardini Montani M.S., Faggioni A., Cirone M. (2017). Bortezomib promotes KHSV and EBV lytic cycle by activating JNK and autophagy. Sci. Rep..

[26] Song C., Mitter S.K., Qi X., Beli E., Rao H.V., Ding J., Ip C.S., Gu H., Akin D., Dunn W.A. (2017). Oxidative stress-mediated NFkappaB phosphorylation upregulates p62/SQSTM1 and promotes retinal pigmented epithelial cell survival through increased autophagy. PLoS ONE.

[27] Granato M., Santarelli R., Filardi M., Gonnella R., Farina A., Torrisi M.R., Faggioni A., Cirone M. (2015). The activation of KSHV lytic cycle blocks autophagy in PEL cells. Autophagy.

[28] Jain A., Lamark T., Sjottem E., Larsen K.B., Awuh J.A., Overvatn A., McMahon M., Hayes J.D., Johansen T. (2010). p62/SQSTM1 is a target gene for transcription factor NRF2 and creates a positive feedback loop by inducing antioxidant response element-driven gene transcription. J. Biol. Chem..

[29] Nezis I.P., Stenmark H. (2012). p62 at the interface of autophagy, oxidative stress signaling, and cancer. Antioxid. Redox Signal..

[30] Jiang T., Harder B., Rojo de la Vega M., Wong P.K., Chapman E., Zhang D.D. (2015). p62 links autophagy and Nrf2 signaling. Free Rad. Biol. Med..

[31] Inman G.J., Binne U.K., Parker G.A., Farrell P.J., Allday M.J. (2001). Activators of the Epstein-Barr virus lytic program concomitantly induce apoptosis, but lytic gene expression protects from cell death. J. Virol..

[32] Cirone M. (2018). EBV and KSHV Infection Dysregulates Autophagy to Optimize Viral Replication, Prevent Immune Recognition and Promote Tumorigenesis. Viruses.

[33] Ye F., Zhou F., Bedolla R.G., Jones T., Lei X., Kang T., Guadalupe M., Gao S.J. (2011). Reactive oxygen species hydrogen peroxide mediates Kaposi’s sarcoma-associated herpesvirus reactivation from latency. PLoS Pathog..

[34] Redza-Dutordoir M., Averill-Bates D.A. (2016). Activation of apoptosis signalling pathways by reactive oxygen species. Biochim. Biophys. Acta.

[35] Trachootham D., Lu W., Ogasawara M.A., Nilsa R.D., Huang P. (2008). Redox regulation of cell survival. Antioxid. Redox Signal..

[36] Perez-Galan P., Roue G., Villamor N., Montserrat E., Campo E., Colomer D. (2006). The proteasome inhibitor bortezomib induces apoptosis in mantle-cell lymphoma through generation of ROS and Noxa activation independent of p53 status. Blood.

[37] Wallington-Beddoe C.T., Pitson S.M. (2017). Enhancing ER stress in myeloma. Aging.

[38] Kumari S., Badana A.K., G M.M., G S., Malla R. (2018). Reactive Oxygen Species: A Key Constituent in Cancer Survival. Biomark. Insights.

[39] Granato M., Gilardini Montani M.S., Santarelli R., D’Orazi G., Faggioni A., Cirone M. (2017). Apigenin, by activating p53 and inhibiting STAT3, modulates the balance between pro-apoptotic and pro-survival pathways to induce PEL cell death. J. Exp. Clin. Cancer Res. CR.

